# Prediction and Measurement of the Damping Ratios of Laminated Polymer Composite Plates

**DOI:** 10.3390/ma13153370

**Published:** 2020-07-29

**Authors:** Hugo Sol, Hubert Rahier, Jun Gu

**Affiliations:** 1 Department Mechanics of Materials and Structures, Vrije Universiteit Brussel, 1050 Brussels, Belgium; 2 Sim vzw, Technologiepark 48, 9052 Zwijnaarde, Belgium; hubert.rahier@vub.be (H.R.); jun.gu@vub.be (J.G.); 3 Physical Chemistry and Polymer Science, Department MACH, Vrije Universiteit Brussel, 1050 Brussels, Belgium

**Keywords:** composite materials, laminated plates, Resonalyser procedure, elastic-viscoelastic correspondence principle, complex stiffness

## Abstract

Laminated composites materials are mostly used in dynamically loaded structures. The design of these structures with finite element packages is focused on vibrations, elastic deformations and failure control. Damping is often neglected because of its assumed secondary importance and also because of dearth of information on relevant material properties. This trend is prone to change as it is now realised that damping plays an increasingly important role in vibration comfort, noise radiation and crash simulations. This paper shows in a first step how to identify the orthotropic elastic and damping properties of single layer fibre-reinforced composite material sheets using a new extended version of the Resonalyser procedure. The procedure is based on the elastic-viscoelastic correspondence principle and uses a mixed numerical experimental method. In a subsequent step, the complex laminate stiffness values are computed using the identified single layer material properties. To validate this approach, the modal damping ratios of arbitrary laminated plates of different materials at several resonance frequencies are predicted and experimentally verified.

## 1. Introduction

Composite materials are increasingly replacing more traditional materials like steel, wood and aluminium in a wide range of applications. From the eighties till the end of the previous century, composite materials were introduced successfully in dynamically loaded structures like satellites, military aircrafts, expensive cars, boats, sporting and many consumer goods. As a result of marked developments and improvements in manufacturing methods, robot assistance and quality control systems, composites are now employed in major structural parts of civil aircrafts and vehicles [1,2]. The success of composites is due to their excellent mechanical properties like high stiffness to weight ratio and high strength to weight ratio, combined with good fatigue resistance. Furthermore, composites offer significant design freedom which allows tailoring of mechanical properties and creation of entirely new shapes. Unfortunately, the mechanics of composites are much more complex than mechanics of traditional isotropic materials. A good introduction to mechanics of composites is given in the book written by Jones [3]. The advancement of composite materials occurred hand in hand with the advancement in computer power and software. In particular, modern finite element software packages are vital in current design of composite structures. Fibre-reinforced composites are mainly used as laminates in dynamically loaded structures. The fundamental building block of laminates is the lamina. A layer in a laminate can be built up with several laminas with the same orientation. Each layer in the laminate can possibly be composed of other materials and may have different orientations and thicknesses. The layers in general exhibit orthotropic material behaviour. In a finite element (FE) model, all layers can be modelled separately. This however requires a huge number of degrees of freedom in the numerical model, which translates to significant increase in memory requirements and computation time. A more economical solution is to represent the laminates by their global laminate stiffness. The computation of the laminate stiffness can be executed at a pre-processing stage using laminate analysis programs.

Finite element analysis of a structure often requires knowledge and input of both the damping behaviour and other relevant material properties into the FE program. Material damping can be defined as ‘the phenomenon of energy dissipation due to inelastic behaviour of a macroscopically uniform material’ Lazan [4]. This definition does not include structural energy dissipation due to, among others, friction between contacting surfaces within a structure, friction in mechanical joints and aerodynamic damping, which are phenomena that are classified under the more general header of ‘structural damping’. An early discussion of modelling and measuring damping was given by Bert [5]. For engineering applications, composite materials like fibre-reinforced plastics are considered as homogeneous from a macroscopic point of view, so that the energy dissipation within the material satisfies the definition of material damping. An early overview of modelling and measuring damping in composite materials was given by Adams [6]. A more recent overview of available literature on damping in composite materials can be found in an article by Treviso et al. [7].

Material damping in fibre-reinforced polymers is a very complex phenomenon. It is due to a variety of contributions which can be distinguished into contributions from the following sources:

- The viscoelastic behaviour of the polymer matrix materials;
- The inelastic behaviour of the fibres;
- The inelastic behaviour of the interphase between fibre and matrix;
- The slip at the fibre/matrix interface in case of non-perfect adhesion;
- Thermo-elastic behaviour of fibres and matrix;
- The volume fractions of matrix and fibre.

Because of the complexity of these different mechanisms, theoretical models to predict quantitatively the material damping of composites are usually cumbersome. If good experimental equipment is available, it is more reliable to measure the damping properties on test specimens. For laminates the damping mechanism becomes even more complex because of the possible delaminations and other interlaminar effects between layers. All methods for predicting the damping of laminates require the damping properties of single layer materials. The quality of the prediction of the entire laminate damping hence relies strongly on the quality of the used single layer material properties.

Many papers to predict laminate damping are based on the elastic-viscoelastic correspondence principle first introduced by Hashin [8,9,10]. According to this elastic-viscoelastic correspondence principle, the formulae for calculation of the complex moduli and stiffness’s of composites are similar as those for the calculation of their elastic counterparts.

In an early paper, Ni and Adams [11] developed a model to predict damping of composite laminates. The experimental values for the layer properties included the elastic and damping values of the longitudinal and transverse Young’s moduli and the shear modulus. These values were measured on clamped beams in flexural and torsional mode shapes. Only the elastic part of Poisson ratio could be measured. The damping part of Poisson ratio was assumed to be zero. All experimental values were measured on composites test beams with a known volume fraction for the matrix and fibre. Micromechanical models were used to predict the elastic and damping values of the same composite materials as the volume fraction varies. Experimental verification was executed on laminated beams in flexural mode shapes. The zero damping of Poisson’s value for the damping was assumed by most authors, since this value cannot be revealed by beam testing. In an effort to overcome this problem, Jong [12] developed a closed form of the solution for the basic damping for Poisson’s ratio. This enabled him to show the influence of the damping part of Poisson ratio on computed laminate damping.

Plates in composite materials are more natural test specimens than beams since they require less preparation and machining and show less influence of stress concentrations and delaminations at the free boundaries. Since analytical solutions for the vibration of orthotropic plates are not available, mixed numerical experimental methods must be used for material identification. In a mixed numerical experimental method, measured vibration quantities like resonance frequencies and damping ratios are compared with similar values computed with a numerical model. The material properties in the numerical model are tuned in such a way that the computed values match the experimental observations. The availability of powerful personal computers has promoted this new approach. The earliest article describing the use of rectangular test plates for the identification of elastic orthotropic moduli of composite material using a mixed numerical experimental method was published by De Wilde and Sol in 1985 [13] and later refined by Sol [14] in 1986. Rayleigh Ritz trial functions were used in the numerical model of rectangular test plates. McIntyre and Woodhouse applied a test procedure on plates to identify not only the elastic but also the damping material properties of composite sheets in 1988 [15]. Other similar early studies were published by Deobald and Gibson [16]. Talbot and Woodhouse [17] proposed a solution for laminate damping by adopting the elastic-viscoelastic correspondence principle, first introduced by Hashin [8]. The laminate damping was computed using classical laminate theory with the same formulas as for the laminate stiffness properties. El Mahi et al. [18] used a finite element model for the identification of elastic and damping values of laminates. They discussed the influence of boundary conditions and concluded that free-free boundary conditions of plates are to be preferred. More recently, Wesolowski and Barkanov [19] also used the elastic-viscoelastic correspondence principle in combination with a mixed numerical experimental method. The paper focused on improving the experiment by eliminating the contribution of air friction to the damping value of the tested laminate. The test was executed in vacuum and the dynamic response was measured contactless with a laser vibrometer. The authors did not take the phase angle of Poisson ratio into account. Marchetti et al. [20] used a laminate model considering linear shear, membrane and bending effects in each layer. The developments used in the paper are based on the analytical multilayer model of Guyader and Lesueur (JSV, 1978). The authors observed good correspondence with experiments in a large frequency band (up to 20 kHz).

A major problem with most of the mixed numerical experimental methods is the necessity to know the correct sequence of mode shapes. To compare measured frequencies and damping ratios with numerical simulations (sometimes with poor starting values), the sequence of computed mode shapes must be the same as in the experiment. This requires the identification of the mode shapes by modal analysis or at least by detecting the position of the nodal lines of the mode shapes. Basic knowledge of modal analysis is described among others in the book by He and Fu [21]. Mode shape measurements and detection of nodal lines are labour intensive and often based on trial and error. Another weak point of mixed numerical methods is that many mode shapes are not sensitive to Poisson’s ratio. Taking more mode shapes of the test plate into account does not solve this problem, since higher mode shapes have increased complex patterns and therefore are more influenced by transverse shear and inertia rotations. This requires numerical thick plate models and additional elastic and damping values associated with transverse shear, which are difficult to identify. The above mentioned problems were solved with the introduction of the Resonalyser method by Sol et al. [22,23]. The Resonalyser method allows fixing the mode shape sequence and assures enough sensitivity to Poisson’s ratio. The Resonalyser method uses simultaneously multiple models including two test beams and a rectangular test plate. The results of the Resonalyser method were intensively compared with values from standard testing by Lauwagie et al. [24] and found to be very accurate. The first applications of the Resonalyser method identified only the elastic orthotropic material properties. De Visscher [25] presented an extension of the Resonalyser method including the identification of the damping properties of orthotropic material properties.

This paper describes as a first step the identification of the complex engineering constants of a single laminate layer. The capabilities of a new and improved version of the Resonalyser method to identify the orthotropic elastic and damping properties of single layer composite material sheets with minimal experimental equipment is demonstrated. Measured resonance frequencies and associated damping ratios of two test beams and a test plate are compared with similar values computed with the finite element method. The orthotropic elastic and damping material properties in the finite element models of the beams and the plate are simultaneously updated in such a way that the computed resonances and damping ratios match all the measured values. This paper introduces for the first time an improvement for the existing Resonalyser method by generating good starting values based on the virtual field method. The virtual field method is described in detail in the book by Pierron and Grediac in [26].

In a next step, the paper shows how to compute the stiffness and damping properties of laminates with the elastic-viscoelastic correspondence principle using previously identified single layer properties. The complex laminate stiffness values are used to predict the modal damping ratio of rectangular plates by finite element simulation. Many experimental values of damping measurements on laminated test beams can be found in literature [6,11,12,20]. Unfortunately, no fully documented experimental values could be found by the authors for damping measurements on test plates.

The described approach to predict laminate damping values is validated experimentally on a test set of laminated plates. The test set includes laminated plates with arbitrary sizes and different composite materials (autoclaved carbon/epoxy, glass/polyester made with hand layup and glass epoxy processed with Resin Transfer Moulding (RTM).

## 2. Identification of the Complex Engineering Constants of Single Layers

### 2.1. Linear Viscoelasticty

Materials like fibre-reinforced polymer composites exhibit viscoelastic behaviour. Viscoelastic behaviour is situated somewhere between pure elastic and pure viscous behaviour. Elastic solids have the capacity to store mechanical energy with no dissipation. Viscous fluids in a non-hydrostatic stress state have the capacity to dissipate energy but cannot store it. Viscoelastic materials can both store and dissipate energy. Most polymers do not show strictly linear viscoelastic behaviour but modelling by the linear viscoelastic theory is a ‘useful starting point’. If the strain history $\varepsilon_{kl}(t)$ is assumed to be continuous and the material is assumed to behave statistically homogeneously, the stress constitutive relation as a function of time $\sigma_{ij}(t)$ can be written as a convolution with a Stieltjes integral in which the integrating function $C_{ijkl}$ is a fourth order material stiffness tensor.

(1) $$\sigma_{ij}(t)=\int_{0}^{\infty }\varepsilon_{kl}(t-s)dC_{ijkl}(s)$$

(Repeated subscripts denote summation over their dimensions). *s* is an arbitrary convolution variable with a dimension of time. Another form of the stress constitutive relation can be found through a change of variable τ=t−s, followed by integration by parts:

(2) $$\sigma_{ij}(t)=\int_{-\infty }^{t}C_{ijkl}(t-\tau )\frac{d\varepsilon_{kl}(\tau )}{d\tau }d\tau$$

Suppose that the material is subjected to a sinusoidal strain with time-independent amplitude $\varepsilon_{kl}^{0}$:

(3) $$\varepsilon_{kl}(t)=\varepsilon_{kl}^{0}e^{i\omega t}$$

ω is the angular frequency and i is the imaginary variable $i=\sqrt{-1}$. The amplitude $\varepsilon_{kl}^{0}$ can be dependent on ω and can be complex. The stress tensor becomes:

(4) $$\sigma_{ij}(t)=i\omega \varepsilon_{kl}^{0}\int_{-\infty }^{t}C_{ijkl}(t-\tau )e^{i\omega \tau }d\tau$$

Again, using the change of variable t−τ=s:

(5) $$\sigma_{ij}(t)=i\omega \varepsilon_{kl}^{0}e^{i\omega t}\int_{0}^{\infty }C_{ijkl}(s)e^{-i\omega s}ds$$

If it is assumed that the sinusoidal strain function is acting on the material for an indefinitely long time so that all initial transient disturbances have died out and assuming that $C_{ijkl}(t)=0$ for *t* < 0, the lower boundary for the integral can be set to −∞ and the integral part can be written as a conventional Fourier integral.

(6) $${\bar{C}}_{ijkl}(\omega )=\int_{-\infty }^{\infty }C_{ijkl}(s)e^{-i\omega s}ds$$

Expression (5) can be written as:

(7) $$\sigma_{ij}(t)=\sigma_{ij}^{0}e^{i\omega t}$$

In which:

(8) $$\sigma_{ij}^{0}(\omega )=i\omega \varepsilon_{kl}^{0}(\omega ){\bar{C}}_{ijkl}(\omega )=C_{ijkl}^{*}\varepsilon_{kl}^{0}(\omega )$$

$C_{ijkl}^{*}=i\omega {\bar{C}}_{ijkl}$ is a complex tensor called the ‘viscoelastic stiffness tensor’.

The stress $\sigma_{ij}(t)$ is hence also a periodic sinusoidal signal with the same angular frequency ω as the strain $\varepsilon_{kl}(t)$. The amplitudes $\varepsilon_{kl}^{0}$ and $\sigma_{ij}^{0}$ are generally complex values and generally not in phase with each other. Because of the symmetry of the stress tensor $\sigma_{ij}^{0}$, the stiffness tensor $C_{ijkl}^{*}$ is *ij* symmetric. Because of the symmetry of the strain tensor $\varepsilon_{kl}^{0}$, the complex stiffness tensor $C_{ijkl}^{*}$ is *kl* symmetric. These symmetries allow contracting the stress tensor $\sigma_{ij}^{0}$ to a column $\sigma_{i}^{*}$, the strain tensor $\varepsilon_{kl}^{0}$ to a column $\varepsilon_{j}^{*}$ and the fourth order stiffness tensor $C_{ijkl}^{*}$ to a second order matrix $C_{ij}^{*}$ by using a contracted notation:
11->1  22->2  33->312->6  13->5  23->4

(9) $$\sigma_{i}^{*}(\omega )=C_{ij}^{*}(\omega )\varepsilon_{j}^{*}\;(i,j\;=1\;\mathrm{to}\;6)$$

$C_{ij}^{*}$ is called the visco-elastic stiffness matrix. Expression (9) is similar to the linear elastic relation between stresses $\sigma_{i}$ and strains $\varepsilon_{j}$ by the elastic stiffness matrix $C_{ij}$. Based on this similarity, Hashin [8] has introduced the so-called ‘elastic-viscoelastic correspondence principle’ of material behaviour of statistically homogeneous fibre-reinforced composite materials. Since the elastic stiffness matrix is *ij* symmetric, the viscoelastic stiffness matrix $C_{ij}^{*}$ is also assumed to be *ij* symmetric. The stress–strain relation (9) and its inverse expression can be written explicitly for a two-dimensional state of plane stress, like it occurs in thin orthotropic composite sheets in a Cartesian axis system (1,2) with the 1- and 2- axis along the orthotropic material axes:

(10) $$\begin{array}{l} \sigma_{i}^{*}=C_{ij}^{*}\varepsilon_{j}^{*}\;or\;inversed\;\varepsilon_{i}^{*}=S_{ij}^{*}\sigma_{j}^{*}\;(i,j=1,2,3)\\ \begin{array}{l} \left\{\begin{array}{c} \sigma_{1}^{*}\\ \sigma_{2}^{*}\\ \tau_{12}^{*} \end{array}\right\}=\left[\begin{array}{ccc} \frac{E_{1}^{*}}{1-\upsilon_{12}^{*}\upsilon_{21}^{*}} & \frac{-\upsilon_{21}^{*}E_{1}^{*}}{1-\upsilon_{12}^{*}\upsilon_{21}^{*}} & 0\\ \frac{-\upsilon_{12}^{*}E_{2}^{*}}{1-\upsilon_{12}^{*}\upsilon_{21}^{*}} & \frac{E_{2}^{*}}{1-\upsilon_{12}^{*}\upsilon_{21}^{*}} & 0\\ 0 & 0 & G_{12}^{*} \end{array}\right]\left\{\begin{array}{c} \varepsilon_{1}^{*}\\ \varepsilon_{2}^{*}\\ \gamma_{12}^{*} \end{array}\right\}\;\;\;or\;\;\;\left\{\begin{array}{c} \varepsilon_{1}^{*}\\ \varepsilon_{2}^{*}\\ \gamma_{12}^{*} \end{array}\right\}=\left[\begin{array}{ccc} \frac{1}{E_{1}^{*}} & -\frac{\upsilon_{21}^{*}}{E_{2}^{*}} & 0\\ -\frac{\upsilon_{12}^{*}}{E_{1}^{*}} & \frac{1}{E_{2}^{*}} & 0\\ 0 & 0 & \frac{1}{G_{12}^{*}} \end{array}\right]\left\{\begin{array}{c} \sigma_{1}^{*}\\ \sigma_{2}^{*}\\ \tau_{12}^{*} \end{array}\right\} \end{array} \end{array}$$

*C* * is the complex in-plane stiffness matrix, S* is the complex in-plane flexibility matrix, $\varepsilon_{1}^{*}$, $\varepsilon_{2}^{*}$ are normal strains and $\sigma_{1}^{*}$, $\sigma_{2}^{*}$ are normal stresses, respectively in the 1- and 2-direction. $\gamma_{12}^{*}$, $\tau_{12}^{*}$ are the in-plane shear strains and stresses. $E_{1}^{*}$, $E_{2}^{*}$ are the complex dynamic Young’s moduli, $\upsilon_{12}^{*}$, $\upsilon_{21}^{*}$ are the major and minor Poisson’s ratios and $G_{12}^{*}$ is the complex in-plane shear modulus. For a given angular frequency ω and the assumed linear behaviour, the values $E_{1}^{*}\;E_{1}^{*}\;\upsilon_{12}^{*}\;\upsilon_{21}^{*}\;G_{12}^{*}$ are constant and called the complex dynamic engineering constants. Because of the symmetry of the relations (10) $\frac{\upsilon_{12}^{*}}{E_{1}^{*}}=\frac{\upsilon_{21}^{*}}{E_{2}^{*}}$ and thus there are only four independent complex engineering constants in the stress–strain relations. The complex engineering constants have a real and an imaginary part as shown in Equation (11):

(11) $$\begin{array}{l} E_{1}^{*}=E_{1}^{\prime }+i.E_{1}^{\prime \prime }=E_{1}^{\prime }(1+i.\tan\delta (E_{1}))\\ E_{2}^{*}=E_{2}^{\prime }+i.E_{2}^{\prime \prime }=E_{2}^{\prime }(1+i.\tan\delta (E_{2}))\\ v_{12}^{*}=v_{12}^{\prime }+i.v_{12}^{\prime \prime }=v_{12}^{\prime }(1+i.\tan\delta (\upsilon_{12}))\\ G_{12}^{*}=G_{12}^{\prime }+i.G_{12}^{\prime \prime }=G_{12}^{\prime }(1+i.\tan\delta (G_{12})) \end{array}$$

The real parts in (11) govern the elastic behaviour and the imaginary ‘tangents delta’ parts govern the damping behaviour of the complex engineering constants. Because of the symmetry relation, if the tangents delta of $E_{1}^{*}$ and $E_{2}^{*}$ are different (which they usually are, since E_1_ is often fibre dominated while *E*_2_ is matrix dominated), Poisson’s ratios $\upsilon_{12}^{*}$ and $\upsilon_{21}^{*}$ cannot simultaneously have a zero tangents delta value.

### 2.2. Identification of the Complex Engineering Constants

A powerful and yet simple method for the experimental identification of both the real and imaginary part of the orthotropic engineering constants is the impulse excitation technique (IET) as described in Heritage et al. [27]. The impulse excitation technique is a non-destructive method to measure resonance frequencies and damping ratios. The procedure consists of tapping the sample with a hammer and recording the induced vibration with an accelerometer, a microphone or a laser Doppler velocity meter. If the test coupon is a beam with a constant cross section, the elastic part of Young’s modulus and shear modulus can be computed from the measured resonance frequency using analytical or empirical formulas. For predefined shapes like rectangular bars, the American Society for Testing Materials (ASTM) E1876 [28] describes how to measure the frequencies and how to compute the complex Young’s and shear modulus.

**Figure 1:**
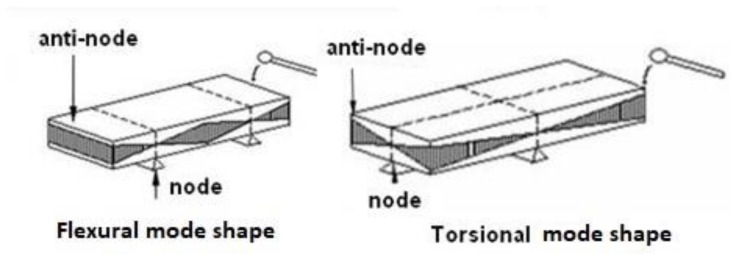
Support, excitation position and measurement positions for impulse excitation technique (IET) tests on beams.

Figure 1 shows how to excite flexural and torsion modal shapes. Specimen support is on the nodal lines (collection of all points with zero vibration amplitude) and the excitation and measurement positions are situated at the anti-nodes (positions with maximal vibration amplitude).

The IET generates (assumed) uni-axial stress fields in the beamlike specimens of Figure 1. This allows computing the Young’s and shear moduli using simple formulas as outlined in the ASTM procedure [28]. If IET is applied on more complex test specimens like plates, more complex states of stress are generated in the test specimen. No ASTM procedures are available, and the simple formulas must be replaced by numerical models of the test specimen. A powerful numerical model is typically a finite element model. An impact in an arbitrary position of a plate causes a vibration pattern that is a superposition of all excited mode shapes. Mode shapes are standing waves vibrating at resonance frequencies. The state of stress generated by an impact is hence complex and varies in time and with the position on the test plate. By measuring the induced vibration at some location and transforming the acquired time domain signal with a mathematical fast Fourier transformation into the frequency domain, a limited number of resonance frequencies of the test plate can be easily detected.

#### 2.2.1. Elastic Part of the Complex Engineering Constants

**Figure 2:**
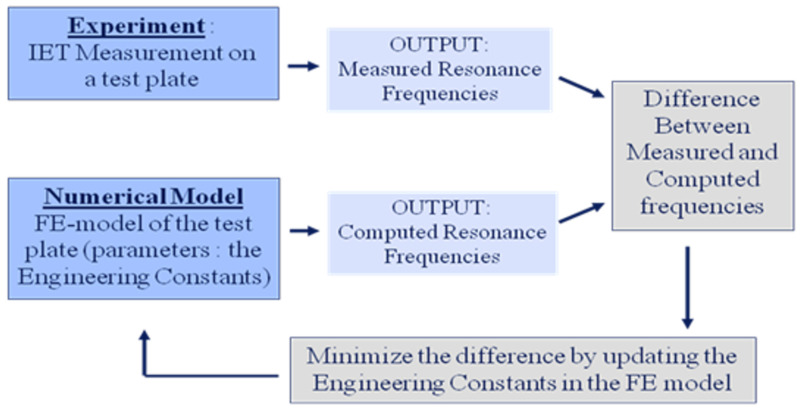
Mixed numerical experimental method: iteratively updating the engineering constants in the numerical finite element (FE) model of a test plate.

Resonance frequencies measured with IET on a test plate allow the identification of the real (thus elastic) part of the complex engineering constants. Resonance frequencies can be computed with the finite element model of the test plate by the solution of an eigenvalue problem as described in He and Fu [21]. The measured resonance frequencies by IET hence can be compared with computed frequencies in a mixed numerical experimental method. Starting with an initial guess of the engineering constants (parameters) in the finite element model, the parameters are iteratively updated till the computed frequencies match the measured frequencies as close as possible (see Figure 2).

Many researchers have used the principle of mixed numerical experimental methods in combination with vibration measurements on test plates in the past [12,13,14,15,16,17,18,19]. All mentioned researchers agreed that free-free boundary conditions were the best configuration for bringing experiment and simulation in agreement. Free-free boundary conditions can be reached by freely suspending the test plate. However, the simple principle shown in Figure 2 suffers from some practical problems. The first problem is that the method requires good starting values for the engineering constants. The computed frequencies with poor initial values can have another mode shape sequence than in the experiment and hence non similar frequencies are compared with each other. This raises the challenge of deciding which computed frequency must be compared with which measured frequency. This problem can be solved by measuring also the mode shapes associated with the resonance frequencies. This however requires much more effort and equipment than just measuring a set of resonance frequencies with IET. A second problem is that an orthotropic plate with an arbitrary aspect ratio of length to width and free-free boundary conditions has mainly mode shapes which are combinations of torsion and bending. The frequencies of such modal shapes are sensitive for Young’s moduli and the shear modulus but not sensitive for Poisson’s ratio. The simultaneous estimation of all the engineering constants during the iterations hence mathematically goes wrong (no convergence to the correct values). Increasing the number of measured resonance frequencies does not solve the problem because the mode shapes associated with higher frequencies have an increasing influence of transverse shear deformations. This compels the replacement of the thin plate model with a thick plate model that requires additional transverse engineering constants, as shown by Frederiksen [29]. Additionally, higher order resonance frequencies are more difficult to measure with IET, especially if the associated damping ratios are high.

**Figure 3:**
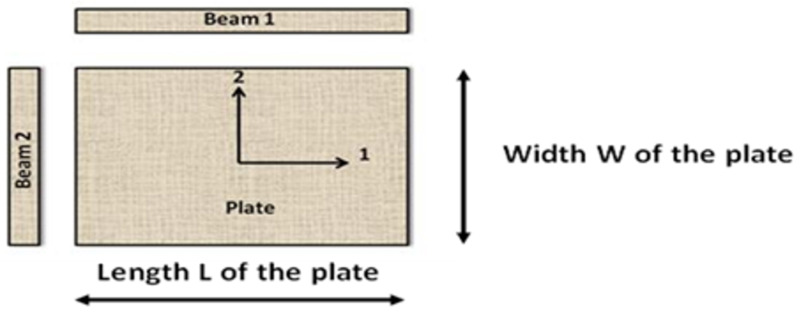
Two test beams cut along the two orthotropic directions, and a test plate with edges parallel to the orthotropic material directions.

The earlier mentioned two challenges can be resolved by extending the number of test specimens in the mixed numerical experimental method. An example of such a multi-specimen method is the ‘Resonalyser’ procedure [22]. The Resonalyser procedure works with three test specimens: two test beams and one test plate. One test beam is cut along the orthotropic material 1-axis, the other beam is cut along the orthotropic material 2-axis (see Figure 3).

A fair value of the Young’s moduli E_1_ and E_2_ can be found with the ASTM IET test on the two test beams. With these values of the Young’s moduli, a special aspect ratio of the test plate can be computed:

(12) $$\frac{L}{W}=\sqrt[{4}]{\frac{E_{1}}{E_{2}}}$$

**Figure 4:**
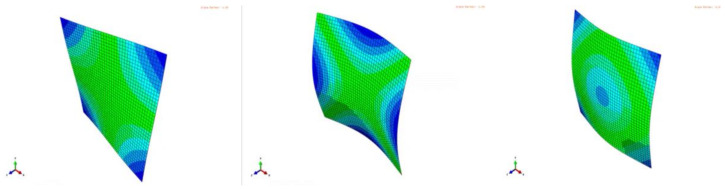
Torsion (left), saddle (middle) and breathing (right) mode shapes of a Poisson plate.

If Poisson’s ratio would be zero, this plate ratio would cause a double bending resonance in the 1- and 2-direction of the plate. A plate with such an aspect ratio is called a ‘Poisson plate’ because the frequencies of the second and third mode shapes are very sensitive for Poisson’s ratio as shown by Sol [14]. The mode shape sequence for a Poisson plate is fixed: first a torsion, next a saddle and third a breathing mode shape (Figure 4).

Hence, without the necessity to do an investigation towards the nature of the mode shapes, an IET on a Poisson plate automatically reveals the three first frequencies and their vibration behaviour. The iterative updating scheme in the mixed numerical/experimental method (Figure 2) can now be applied simultaneously using three finite element models: two beam models and 1 Poisson plate model. The involvement of the finite element models of the two beams and Poisson plate allows conveniently taking the mass of an accelerometer into account as a concentrated nodal mass (if an accelerometer is used for the IET) in the finite element models. However, an experimentalist needs to verify that the Poisson test plate mass is sufficiently large to allow for accurate mass compensation of the combined mass sensor-wires (ratio mass plate / mass sensor at least 200). The final result of the Resonalyser procedure will be an averaged value of all the engineering constants over the two beams and plate area, making the results very suitable as INPUT values for finite element models of composite structures and laminate analysis programs.

The a priori knowledge of the three mode shapes of a Poisson plate together with the knowledge of the basic flexural modal shapes of the two beams allows an additional improvement of the Resonalyser method by adopting the virtual field method. The application of the virtual field method can be used for generating good starting values for all the engineering constants. The principle of the virtual field method starts from the dynamic equilibrium equation of a statistical homogeneous orthotropic thin plate with constant thickness *d* for a free vibration (no external forces) in a Cartesian axis system (*x, y*).

(13) $$D_{11}\frac{\partial^{4}W(x,y,t)}{\partial x^{4}}+D_{22}\frac{\partial^{4}W(x,y,t)}{\partial y^{4}}+2(D_{12}+2D_{66})\frac{\partial^{4}W(x,y,t)}{\partial x^{2}\partial y^{2}}+\rho d\frac{\partial^{2}W(x,y,t)}{\partial t^{2}}=0$$

*W(x, y, t)* is the out of plane transverse displacement, *t* is the time and ρ is the specific mass of the plate material. At this stage, the damping is neglected. In (13),$D_{ij}$ are the orthotropic plate rigidities relating moments *M* to plate curvatures *X* (14):

(14) $$\begin{array}{l} \left\{\begin{array}{c} M_{1}\\ M_{2}\\ M_{12} \end{array}\right\}=\left[\begin{array}{ccc} D_{11} & D_{12} & 0\\ D_{21} & D_{22} & 0\\ 0 & 0 & D_{66} \end{array}\right]\left\{\begin{array}{c} X_{1}\\ X_{2}\\ X_{12} \end{array}\right\} \end{array}\begin{array}{l} D_{11}=\frac{E_{1}d^{3}}{12(1-\nu_{12}\nu_{21})}\\ D_{22}=\frac{E_{2}d^{3}}{12(1-\nu_{12}\nu_{21})}\\ D_{12}=\frac{\nu_{12}E_{2}d^{3}}{12(1-\nu_{12}\nu_{21})}=D_{21}\\ D_{66}=\frac{G_{12}d^{3}}{12} \end{array}\begin{array}{l} X_{1}=\frac{\partial^{2}W}{\partial x^{2}}\\ X_{2}=\frac{\partial^{2}W}{\partial y^{2}}\\ X_{12}=\frac{\partial^{2}W}{\partial x\partial y} \end{array}$$

A general solution of (13) is a sinusoidal vibration with amplitude *w(x, y)* and angular frequency ω. The spatial (time independent) partial differential equation hence becomes:

(15) $$D_{11}\frac{\partial^{4}w(x,y)}{\partial x^{4}}+D_{22}\frac{\partial^{4}w(x,y)}{\partial y^{4}}+2(D_{12}+2D_{66})\frac{\partial^{4}w(x,y)}{\partial x^{2}\partial y^{2}}=\rho d\omega^{2}w(x,y)$$

A finite element solution of (15) involves discretisation of the plate into a mesh of nodal points. The transverse displacement w(x, y) is expressed as a summation of shape functions defined in the nodal points of the FE mesh. This leads to the formulation of set of homogeneous equations:

(16) $$\left[K\right]\left\{U\right\}-\omega^{2}\left[M\right]\left\{U\right\}=\left\{0\right\}$$

$\left[K\right]$ is the assembled plate stiffness matrix, $\left[M\right]$ is the mass matrix and $\left\{U\right\}$ is the column with the displacements in the nodal points of the finite element mesh.

(17) $$K_{ij}=A_{ij}\frac{4bD_{11}}{a^{3}}+B_{ij}\frac{4aD_{22}}{b^{3}}+C_{ij}\frac{4D_{12}}{ab}+E_{ij}\frac{16D_{66}}{ab}$$

(18) $$M_{ij}=H_{ij}\frac{\rho abd}{4}=H_{ij}\frac{Mass\;Plate}{4}$$

*a* is the length and *b* is the width of the plate. The matrices *A, B, C, E* and *H* are finite element connectivity matrices depending on the choice of the shape functions. They are independent of material properties and sizes of the plate. Solutions of (16) can be found by the solution of an eigenvalue problem:

(19) $$\left(\left[K\right]-\lambda \left[M\right]\right)\left\{U\right\}=\left\{0\right\}$$

The solution of the eigenvalue problem yields eigenvalues $\lambda_{i}$ and corresponding eigenvectors ${\left\{U\right\}}_{i}={\left\{\phi \right\}}_{i}$. The three first eigenvectors of the Poisson plate are the mode shapes shown in Figure 4 and *f_i_* is the resonance frequency associated to the mode shapes.

(20) $$\begin{array}{l} \lambda_{i}=\omega_{i}^{2}\\ \omega_{i}=2\pi f_{i} \end{array}$$

Since the nature of the mode shapes of a Poisson plate is known (Figure 4), they can be used as virtual fields. Pre-multiplying with the modal shape of the (i)-th eigenvalue and scaling with the mass matrix yield the Rayleigh quotient:

(21) $${\left\{\Phi \right\}}^{(i)\tau }(\left[K\right]-\lambda^{\left(i\right)}\left[M\right]){\left\{\Phi \right\}}^{\left(i\right)}=\left\{0\right\}$$

(22) $$\mathrm{Yields}:\;\lambda^{\left(i\right)}=\frac{{\left\{\Phi \right\}}^{(i)\tau }\left[K\right]{\left\{\Phi \right\}}^{\left(i\right)}}{{\left\{\Phi \right\}}^{(i)\tau }\left[M\right]{\left\{\Phi \right\}}^{\left(i\right)}}$$

With *(i)* referring to the *(i)*-th eigenvalue and mode shape, following scalar functions *a^(i)^, b^(i)^, c^(i)^* and *e^(i)^* can be defined:

(23) $$\begin{array}{c} a^{\left(i\right)}=\frac{{\left\{\Phi \right\}}_{k}^{\left(i\right)}A_{kl}{\left\{\Phi \right\}}_{l}^{\left(i\right)}}{{\left\{\Phi \right\}}_{k}^{\left(i\right)}H_{kl}{\left\{\Phi \right\}}_{l}^{\left(i\right)}},b^{\left(i\right)}=\frac{{\left\{\Phi \right\}}_{k}^{\left(i\right)}B_{kl}{\left\{\Phi \right\}}_{l}^{\left(i\right)}}{{\left\{\Phi \right\}}_{k}^{\left(i\right)}H_{kl}{\left\{\Phi \right\}}_{l}^{\left(i\right)}},c^{\left(i\right)}=\frac{{\left\{\Phi \right\}}_{k}^{\left(i\right)}C_{kl}{\left\{\Phi \right\}}_{l}^{\left(i\right)}}{{\left\{\Phi \right\}}_{k}^{\left(i\right)}H_{kl}{\left\{\Phi \right\}}_{l}^{\left(i\right)}},e^{\left(i\right)}=\frac{{\left\{\Phi \right\}}_{k}^{\left(i\right)}E_{kl\partial }{\left\{\Phi \right\}}_{l}^{\left(i\right)}}{{\left\{\Phi \right\}}_{k}^{\left(i\right)}H_{kl}{\left\{\Phi \right\}}_{l}^{\left(i\right)}} \end{array}$$

(23) can now be written as:

(24) $$\lambda^{\left(i\right)}=\frac{16b}{a^{3}.Mass}D_{11}a^{\left(i\right)}+\frac{16a}{b^{3}Mass}D_{22}b^{\left(i\right)}+\frac{16}{ab.Mass}D_{12}c^{\left(i\right)}+\frac{64}{ab.Mass}D_{66}e^{\left(i\right)}$$

This equation can be written for the first three mode shapes and eigenvalues of the Poisson plate:

(25) $$\begin{array}{l} \lambda^{\left(Torsion\right)}=\frac{16b}{a^{3}.Mass}D_{11}a^{\left(torsion\right)}+\frac{16a}{b^{3}.Mass}D_{22}b^{\left(torsion\right)}+\frac{16}{ab.Mass}D_{12}c^{\left(torsion\right)}+\frac{64}{ab.Mass}D_{66}e^{\left(torsion\right)}\\ \lambda^{\left(Saddle\right)}=\frac{16b}{a^{3}.Mass}D_{11}a^{\left(Saddle\right)}+\frac{16a}{b^{3}.Mass}D_{22}b^{\left(Saddle\right)}+\frac{16}{ab.Mass}D_{12}c^{\left(Saddle\right)}+\frac{64}{ab.Mass}D_{66}e^{\left(Saddle\right)}\\ \lambda^{\left(Breathing\right)}=\frac{16b}{a^{3}.Mass}D_{11}a^{\left(Breathing\right)}+\frac{16a}{b^{3}.Mass}D_{22}b^{\left(Breathing\right)}+\frac{16}{ab.Mass}D_{12}c^{\left(Breathing\right)}+\frac{64}{ab.Mass}D_{66}e^{\left(Breathing\right)} \end{array}$$

With the associated resonance frequencies of the torsion, saddle and breathing mode shapes measured by IET on the Poisson plate, good values for E_1_ and E_2_ can be found with IET on the beams. By replacing the plate rigidity values *D_ij_* in (25), using the relationship (14) between the plate rigidities and the engineering constants, (25) can be solved for *v_12_* and *G_12_*. Hence the virtual field method provides starting values for all the engineering constants, without the necessity to actually measure the mode shapes. After obtaining the starting values, further identification with the mixed numerical experimental method shown in Figure 2 can be performed using a sensitivity based gradient method as shown by Sol et al. [23].

All the conditions for obtaining good results with the mixed numerical experimental method (Figure 2) are now fulfilled: good starting values, the sequence of mode shapes known, a limited number of frequencies which can easily be measured with IET and the knowledge that the considered resonance frequencies are sensitive for all the engineering constants. The final results will be accurate because it is easy to use very accurate FE models for the beams and the Poisson plate and an IET delivers very accurate values for the measured resonance frequencies, even with non-expensive equipment as shown by Heritage [27].

#### 2.2.2. Identification of the Imaginary Part of the Complex Engineering Constants

**Figure 5:**
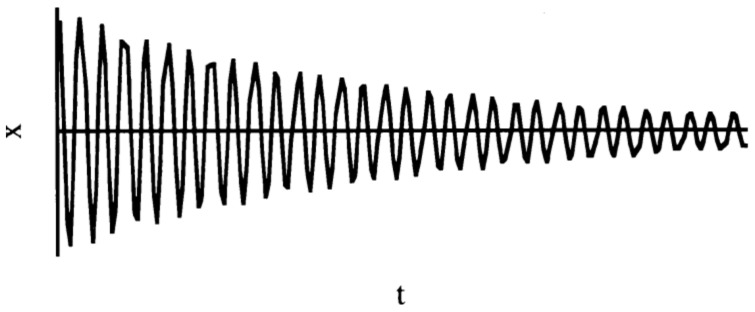
Decaying sinusoidal time domain signal after impact.

The same three test specimens (two beams and 1 Poisson plate), as used for the identification of the elastic part of the engineering constants, are used for identifying the imaginary part. The IET is used again to measure the modal damping ratio associated with the fundamental bending mode shape of the test beams and the modal damping ratios of the three first mode shapes of the Poisson plate. The decaying signal after impact (see Figure 5) is curve fitted in the time domain with the formula:

(26) $$x(t)=X.e^{-\xi \omega t}\sin(\omega t-\varphi )$$

with vibration amplitude $X.e^{-\xi \omega t}$, angular frequency ϖ, phase φ and ξ the modal damping ratio.

**Figure 6:**
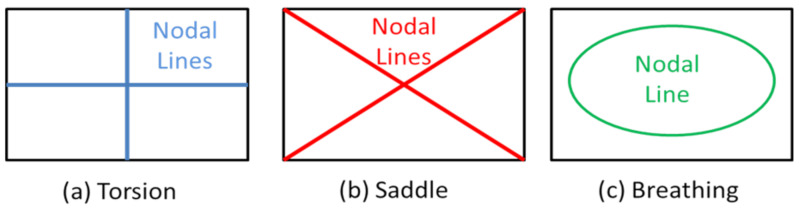
Nodal lines of three mode shapes. (**a**) The nodal lines of the torsion mode shape, (**b**) the nodal lines of the saddle mode shape, (**c**) the nodal line of the breathing mode shape.

Curve fitting (26) for obtaining the modal damping values is only possible if the resonance frequencies can be separated from each other. This is no problem for the fundamental bending resonance frequency of the test beams but requires a special procedure for the measurements of the modal damping ratios of the mode shapes of the Poisson plate. The procedure takes again profit from the special nature of the mode shapes of the Poisson plate (Figure 4). The nodal lines of these three mode shapes are shown in Figure 6. Nodal lines are the collection of points with a zero vibration.

**Figure 7:**
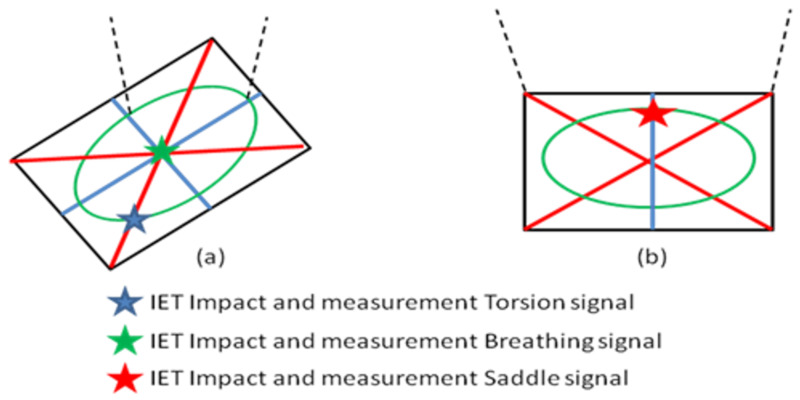
(**a**) Suspension wires fixed in the intersection of the nodal lines of torsion and breathing mode shapes, (**b**) suspension wires fixed in the corners of the Poisson plate.

The suspension wires for the Poisson plate must be fixed on positions on nodal lines of the mode shapes for minimising the influence on the damping ratio measurement with the IET (see Figure 7).

For measuring the decaying signals of the torsion and breathing mode shape after impact, the suspension wires are fixed in the intersection of both their nodal lines (see Figure 7a). The isolated decaying signal of the torsion mode shape is measured by impacting on the intersection of the nodal lines of the saddle and breathing mode shapes (see Figure 7a). The isolated decaying signal of the breathing mode shape is measured by impacting in the centre of the Poisson plate, see Figure 7a. For measuring the decaying signal of the saddle mode shape after impact, the suspension wires are fixed in the corners of the Poisson plate (see Figure 7b). The isolated decaying signal of the saddle mode shape is measured by impacting on the intersection of the nodal lines of the torsion and breathing mode shape (see Figure 7b). In this way the disturbance by the suspension wires is minimised and a decaying signal of only one single mode shape is excited and measured.

The modal damping ratio of a mode shape ξ is proportional to the damping energy *DE* dissipated during one cycle divided by the modal strain energy *PE*.

(27) $$\xi =\frac{DE}{4\pi PE}$$

The strain energy *PE* stored in an elastic body is found by taking half of the volume integral of the contracted product of strain and stress columns:

(28) $$PE=\frac{1}{2}\int_{V}\sigma_{i}^{*}\varepsilon_{i}^{*}dV$$

The modal strain energy can be approximated for sufficiently small damping ratios using only the real part of the in-plane complex stiffness matrix $C_{ij}^{*}$.

(29) $$\left[\begin{array}{ccc} C_{11}^{*} & C_{12}^{*} & 0\\ C_{21}^{*} & C_{22}^{*} & 0\\ 0 & 0 & C_{66}^{*} \end{array}\right]=\left[\begin{array}{ccc} C_{11} & C_{12} & 0\\ C_{21} & C_{22} & 0\\ 0 & 0 & C_{66} \end{array}\right]+i\left[\begin{array}{ccc} C_{11}\tan\delta (C_{11}) & C_{12}\tan\delta (C_{12}) & 0\\ C_{21}\tan\delta (C_{21}) & C_{22}\tan\delta (C_{22}) & 0\\ 0 & 0 & C_{66}\tan\delta (C_{66}) \end{array}\right]$$

(30) $$PE≅\frac{1}{2}\int_{V}C_{ij}\varepsilon_{i}\varepsilon_{j}dV$$

This is acceptable for polymer composite materials where the damping ratio is rarely higher than 1%. For an orthotropic thin plate, taking the symmetry into account, the strain energy *PE* can be expressed as a summation of four different stress–strain combinations:

(31) $$\begin{array}{l} PE=\frac{1}{2}\int_{V}(C_{11}\varepsilon_{1}\varepsilon_{1}+C_{22}\varepsilon_{2}\varepsilon_{2}+2C_{12}\varepsilon_{1}\varepsilon_{2}+C_{66}\gamma_{12}\gamma_{12})dV\\ PE=PE_{11}+PE_{22}+PE_{12}+PE_{66} \end{array}$$

The dissipated energy *DE* during one oscillation cycle is:

(32) $$DE=\int_{V}\left(\oint \sigma_{i}^{*}d\varepsilon_{i}^{*}\right)dV=\int_{V}\left(\oint C_{ij}^{*}\varepsilon_{i}^{*}d\varepsilon_{j}^{*}\right)dV$$

In a similar way as for the strain energy, the dissipated energy *DE* can be expressed as a summation of four different stress–strain combinations:

(33) $$\begin{array}{l} DE=\int_{V}\left(\oint C_{11}^{*}\varepsilon_{1}^{*}d\varepsilon_{1}^{*}+C_{22}^{*}\varepsilon_{2}^{*}d\varepsilon_{2}^{*}+C_{12}^{*}\varepsilon_{1}^{*}d\varepsilon_{2}^{*}+C_{21}^{*}\varepsilon_{2}^{*}d\varepsilon_{1}^{*}+C_{66}^{*}\gamma_{12}^{*}d\gamma_{12}^{*}\right)dV\\ DE=\int_{V}\left(\oint C_{11}^{*}\varepsilon_{1}^{*}d\varepsilon_{1}^{*}+C_{22}^{*}\varepsilon_{2}^{*}d\varepsilon_{2}^{*}+2C_{12}^{*}\varepsilon_{1}^{*}d\varepsilon_{2}^{*}+C_{66}^{*}\gamma_{12}^{*}d\gamma_{12}^{*}\right)dV\\ DE=DE_{11}+DE_{22}+DE_{12}+DE_{66} \end{array}$$

By entering (31) and (33) in (27), De Visscher [25] showed that the modal damping ξ of a mode shape can be written as a summation of contributions from the tangents δ of the stiffness matrix components $C_{ij}^{*}$ weighted with the associated relative modal strain energy portions $\frac{PE_{ij}}{PE}$:

(34) $$\xi =\frac{1}{2}\left(\frac{PE_{11}}{PE}\tan\delta (C_{11})+\frac{PE_{22}}{PE}\tan\delta (C_{22})+\frac{PE_{12}}{PE}\tan\delta (C_{12})+\frac{PE_{66}}{PE}\tan\delta (C_{66})\right)$$

The strain energy portions of (31) can be computed with the finite element model of the Poisson plate. The IET on the test beams and the Poisson plate yields two sets of equations. In the first set (35), the tangents δ of the Young’s moduli are found from the measured damping ratios of the freely suspended beam bending modes.

(35) $$\left\{\begin{array}{c} 2\xi_{Beam1}\\ 2\xi_{Beam2} \end{array}\right\}=\left[\begin{array}{cc} 1 & 0\\ 0 & 1 \end{array}\right]\left\{\begin{array}{c} \tan\delta (E_{1})\\ \tan\delta (E_{2}) \end{array}\right\}$$

The second set (36) yields the tangents δ of the orthotropic stiffness matrix $C_{ij}^{*}$ using the weighting coefficients of expression (34) for the three first plate mode shapes.

(36) $$\left\{\begin{array}{c} 2\xi_{Torsion}\\ 2\xi_{Saddle}\\ 2\xi_{Breathing} \end{array}\right\}=\left[\begin{array}{cccc} GW_{11} & GW_{12} & GW_{13} & GW_{14}\\ GX_{21} & GW_{22} & GW_{23} & GW_{24}\\ GW_{31} & GW_{32} & GW_{33} & GW_{34} \end{array}\right]\left\{\begin{array}{c} \tan\delta (C_{11})\\ \tan\delta (C_{22})\\ \tan\delta (C_{12})\\ \tan\delta (C_{66}) \end{array}\right\}$$

The value of *GW_ij_* in (36) can be found using (34). The simultaneous solution of these two sets of Equations (35) and (36) requires the relation between the tangents δ of the engineering constants and the tangents δ of the stiffness matrix $C_{ij}^{*}$. The relations can be evaluated based on the expression in the complex stiffness matrix (10) and observing that for relatively small damping values tanδ≅δ. For the complex Poisson’s ratios, it can be seen that:

(37) $$\begin{array}{l} \upsilon_{12}^{*}=\frac{C_{21}^{*}}{C_{22}^{*}}=\frac{C_{12}^{*}}{C_{22}^{*}}\;\;\;thus\;\;\;\tan\delta (\upsilon_{12})=\tan\delta (C_{12})-\tan\delta (C_{22})\\ \upsilon_{21}^{*}=\frac{C_{12}^{*}}{C_{11}^{*}}\;\;\;thus\;\;\;\tan\delta (\upsilon_{21})=\tan\delta (C_{12})-\tan\delta (C_{11}) \end{array}$$

The relation for the in-plane shear modulus gives directly:

(38) $$\tan\delta (G_{12})=\tan\delta (C_{66})$$

With the introduction of a complex number $A^{*}=1-\upsilon_{12}^{*}\upsilon_{21}^{*}$ it can be seen in (10) that:

(39) $$\begin{array}{l} \tan\delta (E_{1})=\tan\delta (C_{11})+\tan\delta (A)\\ \tan\delta (E_{2})=\tan\delta (C_{22})+\tan\delta (A) \end{array}$$

The combined set of five Equations (35) and (36) can be solved, taking the relations (37)–(39) into account, resulting in the tangents δ for all the engineering constants.

The combination of the modified Resonalyser method with IET measurements hence provide in a simple way values for the complex engineering constants averaged over the test beams and test plate areas. This makes the results especially suitable for finite element models and laminate analysis programs (as compared with locally identified properties on only beam specimens).

## 3. Identification of Complex Stiffness Values of Laminates

The stress–strain relation (10) in an orthotropic material axis system (1, 2) can be transformed into a stress–strain relation (40) in a rotated axis system (x, y), see Jones [3]

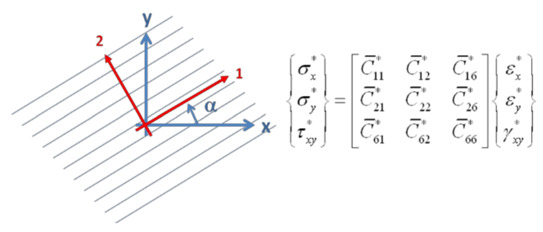
(40)
in which:

(41) $$\begin{array}{l} {\bar{C}}_{11}^{*}=C_{11}^{*}\cos^{4}\alpha +2(C_{12}^{*}+C_{66}^{*})\sin^{2}\alpha \cos^{2}\alpha +C_{22}^{*}\sin^{4}\alpha \\ {\bar{C}}_{12}^{*}=(C_{11}^{*}+C_{22}^{*}-4C_{66}^{*})\sin^{2}\alpha \cos^{2}\alpha +C_{12}^{*}(\sin^{4}\alpha +\cos^{4}\alpha )\\ {\bar{C}}_{22}^{*}=C_{11}^{*}\sin^{4}\alpha +2(C_{12}^{*}+C_{66}^{*})\sin^{2}\alpha \cos^{2}\alpha +C_{22}^{*}\cos^{4}\alpha \\ {\bar{C}}_{16}^{*}=(C_{11}^{*}-C_{12}^{*}-2C_{66}^{*})\sin\alpha \cos^{3}\alpha +(C_{12}^{*}-C_{22}^{*}+2C_{66}^{*})\sin^{3}\alpha \cos\alpha \\ {\bar{C}}_{26}^{*}=(C_{11}^{*}-C_{12}^{*}-2C_{66}^{*})\sin^{3}\alpha \cos\alpha +(C_{12}^{*}-C_{22}^{*}+2C_{66}^{*})\sin\alpha \cos^{3}\alpha \\ {\bar{C}}_{66}^{*}=(C_{11}^{*}+C_{22}^{*}-2C_{12}^{*}-2C_{66}^{*})\sin^{2}\alpha \cos^{2}\alpha +C_{66}^{*}(\sin^{4}\alpha +\cos^{4}\alpha ) \end{array}$$

in which:

(42) $$\begin{array}{l} {\bar{C}}_{11}^{*}=C_{11}(1+i.\tan\delta (C_{11}))\\ {\bar{C}}_{22}^{*}=C_{22}(1+i.\tan\delta (C_{22}))\\ {\bar{C}}_{12}^{*}=C_{12}(1+i.\tan\delta (C_{12}))\\ {\bar{C}}_{66}^{*}=C_{66}(1+i.\tan\delta (C_{66})) \end{array}$$

in which:

(43) $$\begin{array}{l} C_{11}=\frac{E_{1}}{1-\upsilon_{12}\upsilon_{21}}\\ C_{22}=\frac{E_{2}}{1-\upsilon_{12}\upsilon_{21}}\\ C_{12}=\frac{\upsilon_{12}E_{2}}{1-\upsilon_{12}\upsilon_{21}}\\ C_{66}=G_{12}\\ \tan\delta (C_{11})=\tan\delta (E_{1})-\tan\delta (A*)\\ \tan\delta (C_{22})=\tan\delta (E_{2})-\tan\delta (A*)\\ \tan\delta (C_{12})=\tan\delta (\upsilon_{12})+\tan\delta (E_{2})-\tan\delta (A*)\\ \tan\delta (C_{66})=\tan\delta (G_{12}) \end{array}$$

in which the complex number $A^{*}=1-\upsilon_{12}^{*}\upsilon_{21}^{*}$.

**Figure 8:**
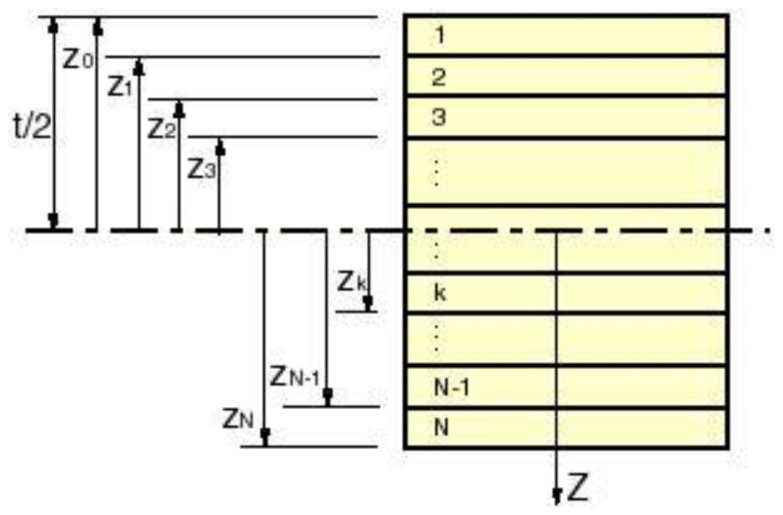
Layer configuration in a laminate.

The classical laminate theory for sufficient thin plates can now be applied to find the complex laminate stiffness values. The laminate is composed of N layers. The z-axis is taken perpendicular to the layer stacking (Figure 8).

The symmetric extensional stiffness matrix A, the symmetric coupling stiffness matrix B and the symmetric bending stiffness matrix D are given by the following expressions:

(44) $$\begin{array}{l} A_{ij}^{*}=\sum_{k=1}^{N}{\left({\bar{C}}_{ij}^{*}\right)}_{k}(z_{k}-z_{k-1})\\ B_{ij}^{*}=\frac{1}{2}\sum_{k=1}^{N}{\left({\bar{C}}_{ij}^{*}\right)}_{k}(z_{k}^{2}-z_{k-1}^{2})\\ D_{ij}^{*}=\frac{1}{3}\sum_{k=1}^{N}{\left({\bar{C}}_{ij}^{*}\right)}_{k}(z_{k}^{3}-z_{k-1}^{3}) \end{array}$$

## 4. Computation of the Modal Damping Ratio of Laminated Plates

Plates with a symmetric laminate configuration have no coupling between the in-plane rigidities A and the plate rigidities *D*. The transverse vibration mode shapes of a symmetric plate are hence dominated only by the plate rigidities. Making use of the definitions of the bending and torsion moments, it is possible to express the moments as a function of the global plate curvatures:

(45) $$\left\{\begin{array}{c} M_{x}^{*}\\ M_{y}^{*}\\ M_{xy}^{*} \end{array}\right\}=\left[\begin{array}{ccc} D_{11}^{*} & D_{22}^{*} & D_{16}^{*}\\ D_{21}^{*} & D_{22}^{*} & D_{26}^{*}\\ D_{61}^{*} & D_{62}^{*} & D_{66}^{*} \end{array}\right]\left\{\begin{array}{c} \chi_{x}^{*}\\ \chi_{y}^{*}\\ \chi_{xy}^{*} \end{array}\right\}$$

$M_{x}^{*},M_{y}^{*}\;\mathrm{and}\;\chi_{x}^{*},\chi_{y}^{*}$ are complex bending moments and curvatures, $M_{xy}^{*}\;\mathrm{and}\;\chi_{xy}^{*}$ are the complex torsion moment and the complex torsion curvature and $D_{ij}^{*}=D_{ij}(1+i.\tan\delta (D_{ij}))$. The total strain energy *PE* of an anisotropic vibrating thin plate can be expressed as:

(46) $$\begin{array}{l} PE=\frac{1}{2}\int_{S}\left[D_{11}X_{x}^{2}+D_{22}X_{y}^{2}+D_{66}X_{xy}^{2}+2D_{12}X_{x}X_{y}+2D_{16}X_{x}X_{xy}+2D_{26}X_{y}X_{xy}\right]dS\\ PE=PE_{11}+PE_{22}+PE_{66}+PE_{12}+PE_{16}+PE_{26} \end{array}$$

*PE_ij_* is partial portions related to the anisotropic plate rigidities *D_ij_*. The modal damping ratio of a mode shape of a laminated plate can be computed similar as (34):

(47) $$\xi =\frac{1}{2}\left(\frac{PE_{11}}{PE}\tan\delta (D_{11})+\frac{PE_{22}}{PE}\tan\delta (D_{22})+\frac{PE_{66}}{PE}\tan\delta (D_{66})+\frac{PE_{12}}{PE}\tan\delta (D_{12})+\frac{PE_{16}}{PE}\tan\delta (D_{16})+\frac{PE_{26}}{PE}\tan\delta (D_{26})\right)$$

The total strain energy *PE* and the partial strain energy portions *PE_i_*_j_ related to the plate rigidities can be calculated by the FE model of the plate.

## 5. Experiments and Validations

### 5.1. Experiment 1: Laminated Carbon/Epoxy Plates

An UD 40 cm × 30 cm carbon/epoxy plate with all ten layers oriented in the 0° direction was prepared in an autoclave, according to the suppliers instructions (2-h de-bulking under vacuum at room temperature, 2 h in autoclave at 20 mbar vacuum and 4 bar pressure, curing temperature 125 °C). The layers were cut out of a 30 cm width bobbin CMP 200/300 CP0031 carbon/epoxy prepreg material. This UD test plate was used in a first step to identify the single layer orthotropic properties of the material using the extended Resonalyser procedure.

Two test beams were cut respectively along the fibre direction and perpendicular to the fibre direction of the UD plate. Table 1 shows the sizes and masses of the two beams. The test beams were freely suspended on thin nylon wires and the resonance frequencies were measured using IET with a small wooden hammer and a micro 0.00025 accelerometer DJB Instruments type A/128/V1 with a sensitivity of 5.26 mV/g. The sizes, mass and IET test results of the beams are shown in Table 1 and Table 2.

The IET vibration results of the two carbon/epoxy beams are given in Table 2.

A Poisson plate with aspect ratio according to formula (12) was cut out of the UD carbon/epoxy plate. The sizes and mass of the Poisson plate are given in Table 3.

The resonance frequencies of the Poisson plate, measured with the IET are given in Table 4.

The virtual field method uses the resonance frequencies of the beams and Poisson plate to generate good starting values (using typical torsion, saddle and breathing mode shapes, preliminarily stored on computer disk) for the elastic part of the engineering constants. Next, the mixed numerical experimental method as described in 2.2.1 computes iteratively the final elastic part of the engineering constants:

- E_1__Start = 1.125E + 11 [Pa]            E_1__Final = 1.092E + 11 [Pa]
- E_2__Start = 7.540E + 09 [Pa]            E_2__Final = 7.303E + 09 [Pa]
- v_12__Start = 4.907E - 01 [-]                v_12__Final = 0.476 [-]
- G_12__Start = 3.188E + 09 [Pa]          G_12__Final = 3.660E + 09 [Pa]

**Figure 9:**
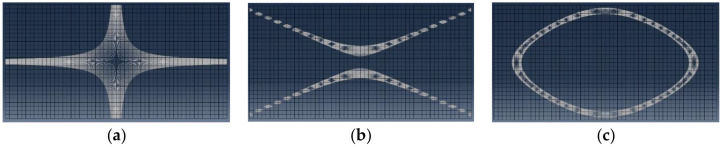
Nodal lines of the mode shapes of the Poisson plate, computed with the FE model. (**a**) Torsion mode shape, (**b**) saddle mode shape and (**c**) breathing mode shape.

With these elastic values of the engineering constants, the FE model of the Poisson plate can plot the nodal lines of the three first mode shapes (see Figure 9).

**Figure 10:**
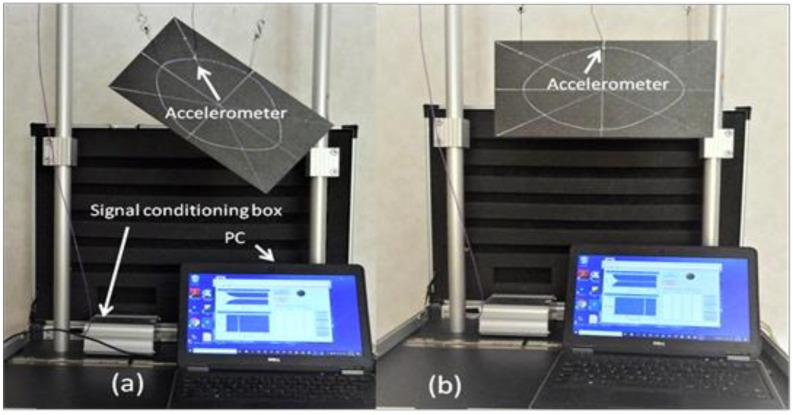
Test setup for measuring the damping ratios with IET: An aluminium suspension frame and the DJB micro accelerometer connected through a signal conditioning box with a PC; in (**a**) the accelerometer is fixed with beeswax on the intersection of the nodal lines of the saddle and breathing mode shapes; in (**b**) the accelerometer is fixed on the intersection of torsion and breathing mode shape.

The knowledge of these nodal lines allows finding the correct position of the intersection points to measure the damping ratios of the beams and Poisson plate using IET. Figure 10a shows the test setup for the measurement of the damping ratios of the torsion and breathing mode shapes and Figure 10b shows the suspended Poisson plate ready for the measurement of the saddle mode shape.

**Figure 11:**
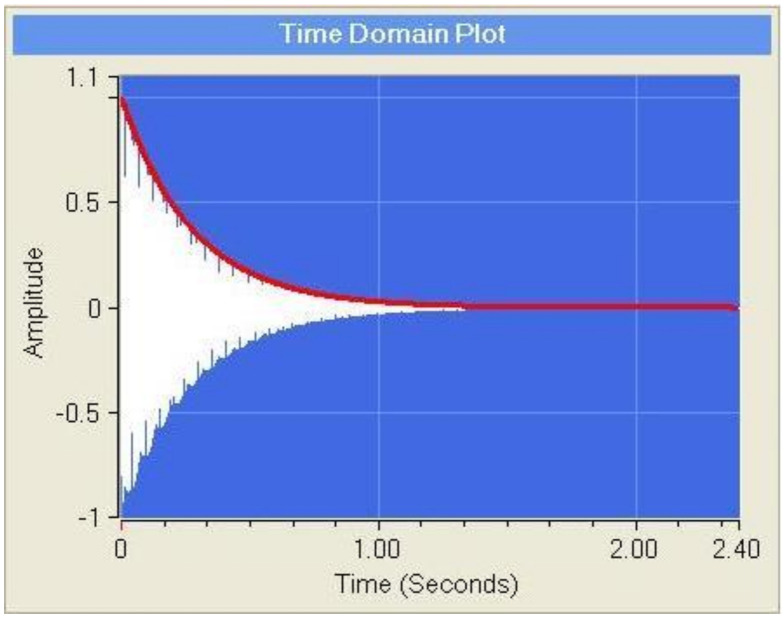
Curve fitted decaying sinusoidal signal. The white zone is the (compressed) recorded sinusoidal signal; the red envelope line is the curve fitted damped exponential function of the signal. The example shows a signal with resonance frequency = 241.2 Hz and a damping ratio value of 0.00237.

The measured decaying sinusoidal signal can be curve fitted with the formula (26). A curve fitting example is shown in Figure 11.

It can be seen that this apparently very low value for the damping ratio is able to damp out the signal completely in about 1 s. The obtained modal damping ratios by curve fitting of the measured signals with IET for the Poisson plate and the test beams are given in Table 5 together with their resonance frequencies.

All experimental information is now available to compute the complex engineering constants with the procedure described in 2.2.2. The result is shown in Table 6.

With these single layer properties, the damping ratio of arbitrary laminated plates with arbitrary aspect ratios can be predicted using the classical laminate theory and the elastic-viscoelastic correspondence principle.

**Figure 12:**
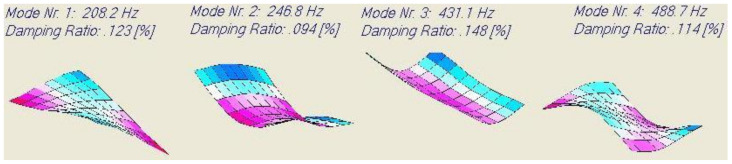
Computed resonance frequencies, mode shapes and damping ratios of a carbon/epoxy (0.2365 m × 0.1385 m × 0.0021 m) laminated plate with stacking sequence (0° −45° 45° −45° 0°)_S._

A 40 cm × 30 cm ten-layer symmetric laminate (0° −45° 45° −45° 0°)_S_ was prepared in an autoclave. The layers were cut out of the 30 cm width bobbin CMP 200/300 CP0031 carbon/epoxy prepreg material, the same as used for the single layer test. The measured average thickness of this laminate was 2.1 mm and the computed complex anisotropic plate rigidities of this laminated plate are shown in Table 7:

The computed resonance frequencies, mode shapes and damping ratios for a (0.2365 m × 0.1385 m × 0.0021 m) and a mass of 0.1011 kg laminated test plate using a thin plate finite element model are shown in Figure 12.

These predicted values were compared with experimental measurements. The test plate was freely suspended, and the resonance frequencies were measured with IET and the DJB A/128/V1 micro accelerometer. Because the exact positions of all the nodal lines of the mode shapes of this laminated plate are not precisely known, the test plate could not be suspended with the nylon strings for each mode shape positioned on a nodal line. Also, the position of the hammer impact and the measurement position of the accelerometer required some trial and error. The measurement of the damping ratio was not possible with IET for the same reasons and additionally because the mode shapes could not be excited separately by a hammer impact. Therefore, to measure the damping ratios, acoustic excitation by a small Bluetooth loudspeaker (Philips model BT55B/00) was used. With a frequency generator, an acoustic sinusoidal signal with the targeted resonance frequency was sent as excitation signal and after cutting the excitation, the freely decaying signal was captured by the DJB A/128/V1 accelerometer. The experimental values are compared with the numerical predictions in Table 8.

It can be observed in Table 8 that the measured damping ratios are consistently higher than the predicted values. This can be explained by the influence of the suspension wires and the (although very tiny) cable of the accelerometer on a 0.1011 kg light test plate. The measured damping can possibly also have additional contributions from interlaminar effects and transverse shear deformations, but these assumptions could not be investigated at this stage. Because the position of the nodal line is well-known for a beam in a first bending mode, it is easier to measure the damping correctly on beam specimens. Therefore a (0.188m × 0.02565m × 0.0021m) beam was cut out of the laminated plate in the X- direction. The predicted and measured results are shown in Table 9.

### 5.2. Experiment 2: Polyester Reinforced with UD Glass Fabric

A 40 cm × 40 cm glass/polyester plate with eight UD glass textile layers of 600g/m^2^ oriented in the 0° direction was prepared with hand layup, cured and compressed between two thick aluminium plates. The cured plate had a final thickness of 4.54 mm. The same measurement procedure for obtaining the single layer properties described in Experiment 1 is applied. The size and mass of the two test beams and the resulting Poisson plate are given in Table 10.

Table 11 gives the results of the same measurement procedure for the elastic and damping part as described in Experiment 1. The difference between the stiffness values of *E_1_* and *E_2_* are not as extreme as for the UD carbon epoxy. The tangents delta values are higher.

**Figure 13:**
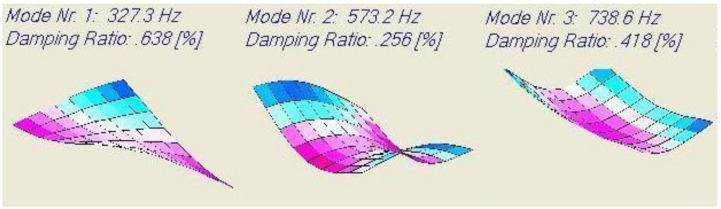
Computed resonance frequencies, mode shapes and damping ratios of a carbon/epoxy.

With these single layer properties, the damping ratio of a test plate cut under an angle of 20° of the Poisson plate, is predicted and measured. The dimensions of the test plate are (0.177 m × 0.1337 m × 0.00454 m) and the mass is 0.19658 kg. The computed complex anisotropic plate rigidities of this glass/polyester plate are shown in Table 12:

The computed resonance frequencies, mode shapes and damping ratios for the 20° glass/polyester test plate using a thin plate finite element model are shown in Figure 13.

Only three frequencies could be measured by the available IET and the DJB accelerometer. The associated damping ratios were identified using acoustic excitation with the Bluetooth loudspeaker. Table 13 shows the results.

The glass/polyester plate was not laminated (excluding interlaminar effects) and was heavier than the previously tested carbon/epoxy plate (reducing the influence of wires). The measured values are however again higher than the predicted values. To further investigate this problem, a (0.129 m × 0.0217 m × 0.00454 m) beam in the x-direction was cut out of the plate.

The predicted value for the beam in Table 14 is again lower than the measured value. The probable explanation is that the test plate as well as the beam has a large thickness to length ratio and hence the transverse shear can possibly increase the measured damping ratio value. The length to thickness ratio of the test plate was 39 and the length to thickness ratio of the beam was 28. To investigate the possibility of transverse shear influence, in the third example a thinner and heavier test plate is investigated.

### 5.3. Experiment 3: Epoxy Reinforced with UD Glass Fabric

A 40 cm × 40 cm glass/polyester plate with three UD glass textile layers of 600 g/m2 oriented in the 0° direction was fabricated with an RTM manufacturing technique. The textile layers were separated by chopped strand mats of E glass of 225 g/m2. The same measurement procedure for obtaining the single layer properties as described in Experiments 1 and 2 is applied. The ‘single layer’ properties must now be regarded as the average over the thickness of the RTM layers. The obtained engineering constants are hence the apparent values in bending over the thickness of the test plate. The size and mass of the two test beams and the resulting Poisson plate are given in Table 15.

Table 16 gives the results of the same measurement procedure for the elastic and damping part as described in Experiment 1 and 2. The difference between the stiffness values of E_1_ and E_2_ are not as large as for the UD carbon epoxy. The tangents delta values are higher than for the UD carbon epoxy and the glass polyester plates in Experiments 1 and 2.

**Figure 14:**
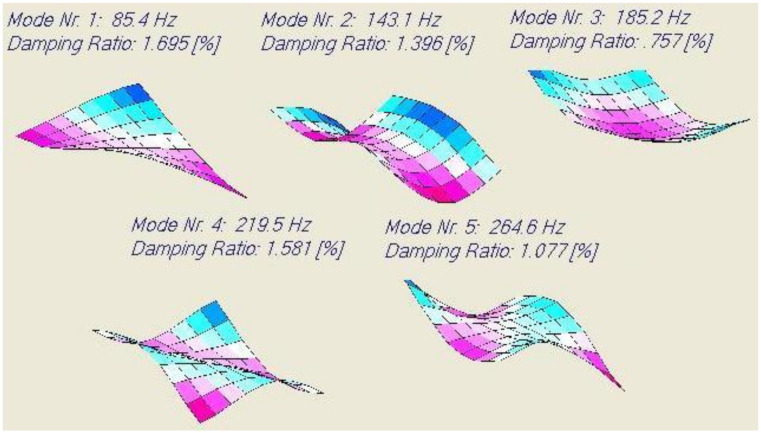
Computed resonance frequencies, mode shapes and damping ratios of a carbon/epoxy.

With these single layer properties, the damping ratio of a test plate cut at an angle of 20° is predicted and measured. The dimensions of the test plate are (0.275 m × 0.235 m × 0.0042 m) and the mass is 0.3731 kg. The length to thickness ratio of the test plate is 65, which is closer to the thin plate assumptions of the test plate than in experiment 2. The computed complex anisotropic plate rigidities of this glass/polyester plate are shown in Table 17:

The computed resonance frequencies, mode shapes and damping ratios for the 20° glass/epoxy test plate using a thin plate finite element model are shown in Figure 14. It can be observed that the damping values of the test plate are higher than in the previous two examples.

Five resonance frequencies were measured using IET and the DJB accelerometer. The associated damping ratios were identified using acoustic excitation with the Bluetooth loudspeaker. Table 18 shows the comparison between the predicted and measured results.

The measured damping values are now closer to the predicted values than in the previous two examples. This can be explained because the mass of the test plate was now higher than in the previous cases (reducing the influence of wires) and the length to thickness ratio was higher (reducing the influence of transverse shear deformations). It is hence recommended that the ratio width/thickness of the Poisson plate is higher than 40.

Measuring the decaying signal with a non-contacting laser Doppler velocity meter could partially solve the problem of additional structural damping contributions of accelerometer cables, but unfortunately a laser Doppler velocity meter is 20 times more expensive than a micro accelerometer and much more difficult to handle on freely suspended test plates (rigid body oscillations after impact).

## 6. Conclusions

Measuring damping is much more difficult than measuring resonance frequencies. The physical reason for this fact is that in one vibration cycle the absorbed damping energy is very small (order of magnitude 1%) as compared with the strain energy. This paper showed in the first step, how to identify the orthotropic elastic and damping properties of the single layer fibre-reinforced composite material sheets using a new mixed numerical experimental method, which is an extension of the Resonalyser procedure. The extended procedure allows generating good starting values based on the virtual fields method. The procedure also takes the influence of the mass and wires of used accelerometers into account through corrections in the finite element model. However, the experimentalist needs to verify that the Poisson test plate mass is sufficiently large to allow for accurate mass compensation of the combined mass sensor-wires (ratio mass plate/mass sensor at least 200). Additionally, the value of the ratio width/thickness of the used Poisson test plates must be sufficiently high to limit the influence of transverse shear and rotation inertia in the used thin plate model (ratio width/thickness at least 40). With these precautions, the new extended Resonalyser procedure can identify accurately all the orthotropic elastic and damping properties of single layer fibre-reinforced composite material sheets. The single layer properties of three different composite materials are identified as examples. Table 6 shows the identified single layer orthotropic complex moduli of carbon/epoxy. Table 11 and Table 16 show similar values of glass/epoxy and glass/polyester material. It can be observed that the phase angle of Poisson ratio in all the examples is found to be different from zero. In a subsequent step, the paper shows how complex laminate stiffness values can be computed with finite element software using the identified single layer material properties. To validate this approach, the modal damping ratios at several resonance frequencies of laminated plates of different materials and with arbitrary sizes and arbitrary laminate configurations are first predicted with FE simulations and next experimentally verified. The paper showed that the proposed extended Resonalyser procedure allows successful identification of averaged values of all the in-plane complex engineering constants of composite material sheets and the prediction of laminate damping of plates with only using relatively simple equipment.

## Figures and Tables

**Table 1:** Sizes and mass of the two carbon/epoxy test beams.

Test Specimen	Length [m]	Width [m]	Thickness [m]	Mass [kg]
Beam 1 (0°)	3.550E-01	1.840E-02	2.080E-03	1.995E-02
Beam 2 (90°)	2.320E-01	2.400E-02	2.060E-03	1.701E-02

**Table 2:** IET test results of the two carbon/epoxy test beams.

IET Test Specimen	Frequency [Hz]	Damping Ratio [%]
Beam 1 (0°)	146.5	0.037
Beam 2 (90°)	87.5	0.372

**Table 3:** Size and mass of the carbon/epoxy Poisson plate.

Test Specimen	Length [m]	Width [m]	Thickness [m]	Mass [kg]
Poisson plate	2.710E-01	1.390E-01	2.120E-03	1.174E-01

**Table 4:** IET frequency test results of the Poisson plate.

Mode Shape Type	Frequency [Hz]
Torsion	94.4
Saddle	241.2
Breathing	264.4

**Table 5:** IET frequency and damping test results of the Poisson plate.

Mode Shape Type	Frequency [Hz]	Damping Ratio [%]
Torsion	94.4	0.500
Saddle	241.2	0.237
Breathing	264.4	0.174

**Table 6:** Identified single layer orthotropic complex moduli of carbon/epoxy.

Engineering Constant	Real Part [GPa]	Imaginary Part [GPa]	Tangents Delta [-]
Young’s Modulus E_1_	109.2	8.1E-02	0.00074
Young’s Modulus E_2_	7.303	5.4E-02	0.00744
Major Poisson’s ratio v_12_	0.476	−1.4E-03	−0.00294
Minor Poisson’s ratio v_21_	0.032	1.2E-04	0.00376
In-plane Shear Modulus G_12_	3.660	3.7E-02	0.01021

**Table 7:** Computed complex anisotropic plate rigidities.

Plate Rigidity	Real Part [Nm]	Imaginary Part [Nm]
D_XX_	56.06	0.0631
D_YY_	16.45	0.0523
D_XY_	12.12	0.0081
D_ZZ_	12.22	0.0247
D_XZ_	−3.99	−0.001
D_YZ_	−3.99	−0.001

**Table 8:** Test results of the (0° −45° 45° −45° 0°)_S_ carbon/epoxy laminate plate.

Mode	Predicted Frequency [Hz]	Measured Frequency [Hz]	Predicted Damping Ratio [%]	Measured Damping Ratio [%]	Difference Predict-Measured [%]
1	208.2	205.9	0.123	0.147	0.024
2	246.8	244.3	0.094	0.103	0.009
3	431.1	431.0	0.148	0.177	0.029
4	488.7	481.0	0.114	0.142	0.028

**Table 9:** Test results of the (0° −45° 45° −45° 0°)_S_ carbon/epoxy laminate beam.

Mode	Predicted Frequency [Hz]	Measured Frequency [Hz]	Predicted Damping Ratio [%]	Measured Damping Ratio [%]
Beam-X	394	394.5	0.082	0.082

**Table 10:** Size and mass of the glass/polyester test specimens.

Test Specimen	Length [m]	Width [m]	Thickness [m]	Mass [kg]
Beam 1 (0°)	2.91E-01	2.44E-02	4.5E-03	5.874E-02
Beam 2 (90°)	2.11E-01	2.33E-02	4.5E-03	4.059E-02
Poisson plate	2.4E-01	1.95E-01	4.54E-03	3.9057E-01

**Table 11:** Identified single layer orthotropic complex moduli of glass/polyester.

Engineering Constant	Real Part [GPa]	Imaginary Part [GPa]	Tangents Delta [-]
Young’s Modulus E_1_	32.7	8.5E-02	0.0026
Young’s Modulus E_2_	13.7	1.36E-01	0.0099
Major Poisson’s ratio v_12_	0.27	−1.4E-03	−0.00506
Minor Poisson’s ratio v_21_	0.11	2.46E-04	0.00224
In-plane Shear Modulus G_12_	4.27	5.91E-02	0.01384

**Table 12:** Computed complex anisotropic plate rigidities of the glass polyester plate.

Plate Rigidity	Real Part [Nm]	Imaginary Part [Nm]
D_XX_	226.5	0.75
D_YY_	109.6	1.07
D_XY_	48.48	0.10
D_ZZ_	51.96	0.42
D_XZ_	46.76	−0.12
D_YZ_	2.28	−0.021

**Table 13:** Test results of the 20° glass/polyester plate.

Mode	Predicted Frequency [Hz]	Measured Frequency [Hz]	Predicted Damping Ratio [%]	Measured Damping Ratio [%]	Difference Predict-Measured [%]
1	327.3	327	0.638	0.680	0.042
2	573.2	574	0.256	0.288	0.032
3	738.6	736	0.418	0.502	0.086

**Table 14:** Test results of the glass/polyester 20° beam.

Mode	Predicted Frequency [Hz]	Measured Frequency [Hz]	Predicted Damping Ratio [%]	Measured Damping Ratio [%]	Difference Predict-Measured [%]
Beam	944.8	946	0.424	0.527	0.103

**Table 15:** Size and mass of the glass/epoxy RTM test specimens.

Test Specimen	Length [m]	Width [m]	Thickness [m]	Mass [kg]
Beam 1 (0°)	1.62E-01	1.885E-02	3.83E-03	1.615E-02
Beam 2 (90°)	1.26E-01	2.84E-02	4.22E-03	2.073E-02
Poisson plate	0.15E-01	1.2E-01	3.81E-03	9.436E-02

**Table 16:** Identified orthotropic complex moduli of the glass/epoxy Poisson plate.

Engineering Constant	Real Part [GPa]	Imaginary Part [GPa]	Tangents Delta [-]
Young’s Modulus E_1_	15.1	1.30E-01	0.00864
Young’s Modulus E_2_	5.7	1.76E-01	0.03094
Major Poisson’s ratio v_12_	0.33	−1.1E-03	−0.00335
Minor Poisson’s ratio v_21_	0.13	5.5E-03	0.0429
In-plane Shear Modulus G_12_	1.9	6.93E-02	0.03648

**Table 17:** Computed complex anisotropic plate rigidities of the glass/epoxy plate.

Plate Rigidity	Real Part [Nm]	Imaginary Part [Nm]
D_XX_	79.94	0.92
D_YY_	34.17	1.07
D_XY_	16.60	0.26
D_ZZ_	17.71	0.39
D_XZ_	16.73	−0.08
D_YZ_	2.47	0.02

**Table 18:** Test results of the 20° glass/polyester plate.

Mode	Predicted Frequency [Hz]	Measured Frequency [Hz]	Predicted Damping Ratio [%]	Measured Damping Ratio [%]	Difference Predict-Measured
1	85.4	83	1.695	1.741	0.046
2	143.1	143	1.396	1.418	0.022
3	185.2	182	0.757	0.801	0.044
4	219.5	221	1.581	1.641	0.060
5	264.6	257	1.077	1.144	0.063
